# Clinical Variants, Characteristics, and Outcomes Among COVID-19 Patients: A Case Series Analysis at a Tertiary Care Hospital in Karachi, Pakistan

**DOI:** 10.7759/cureus.14761

**Published:** 2021-04-29

**Authors:** Tasnim Ahsan, Bharta Rani, Roomana Siddiqui, Glenis D‘Souza, Razzaq Memon, Irfan Lutfi, Omer I. Hasan, Rushma Javed, Farhan Khan, Muhammad Hassan

**Affiliations:** 1 Internal Medicine: Diabetes & Endocrinology, Jinnah Postgraduate Medical Centre, Karachi, PAK; 2 Internal Medicine, Diabetes and Endocrinology, Jinnah Postgraduate Medical Centre, Medicell Institute of Diabetes Endocrinology & Metabolism (MIDEM), Karachi, PAK; 3 Internal Medicine, OMI Institute, Karachi, PAK; 4 Interventional Radiology, Shaheed Mohtarma Benazir Bhutto Medical College, Dow University of Health Sciences, Karachi, PAK

**Keywords:** disease mortality, severity, clinical markers, predictors, pakistan, covid-19

## Abstract

Introduction

Coronavirus disease 2019 (COVID-19) has become a global threat to public health. The current study investigates alterations in the biological estimates concerning the severity, recovery, mortality, and assessment of treatment-based outcomes.

Methods

A case series of 165 COVID-19 patients admitted to OMI Institute (a tertiary care hospital) was conducted between May and August 2020. The data regarding demographic characteristics, comorbid conditions, radiographic abnormalities, biological estimations, symptoms, treatment, disease progression, complications, and outcomes were recorded using a structured questionnaire. Laboratory estimations included complete blood count (CBC), renal and electrolyte profile, liver function tests (LFTs), hematological indices, and inflammatory markers. Chest X-ray, electrocardiogram (ECG), and a high-resolution computed tomography (HRCT) scan were also performed, and data were extracted from the medical records. Analysis was done using the Statistical Package for the Social Sciences (SPSS) version 22.0.

Results

Out of the 165 COVID-19 patients, 79.4% recovered and were successfully discharged, while 20.6% of inpatient died. The patients' mean age was 56.03 ± 15.96 years, with a male majority (55.1%). The most common comorbid conditions were diabetes and hypertension; fever and dry cough were among the most frequently reported symptoms. The chest imaging findings among the severe/critical COVID-19 patients showed extensive bilateral patchy opacities. The median laboratory investigations, including neutrophil-to-lymphocyte ratio (NLR) (14.83), C-reactive protein (CRP) (7.4 mg/dl), lactate dehydrogenase (LDH) (786 IU/L), ferritin (1401.15 mcg/ml), and mean oxygen saturation (88.25%), were significantly altered among cases with increased disease severity and those who expired (p<0.05). The proportion of acute respiratory distress syndrome (ARDS) and sepsis development was significantly high among severe/critical COVID-19 patients (p<0.05). Treatment with tocilizumab, remdesivir, doxycycline, ivermectin, enoxaparin sodium, and steroids was deemed to be potentially effective treatment options in terms of reducing COVID-19 severity and chances of recovery. Furthermore, age (OR 1.05; p=0.047), presence of comorbidity (OR 8.471; p=0.004), high NLR, LDH (final outcome) (OR 1.361 and 1.018; p<0.05), and CRP levels (midpoint) (OR 1.631; p=0.05) were identified as the strong predictors of death among COVID-19 patients.

Conclusion

The study identified several alterations in the clinical profile of the COVID-19 patients concerning severity during the hospital stay, affecting prognosis. Clinically, tocilizumab, remdesivir, doxycycline, ivermectin, enoxaparin sodium, and steroids were identified as potential therapeutic options for COVID-19 due to their ability to alter disease-associated severity and recovery rate.

## Introduction

The novel coronavirus disease 2019 (COVID-19) is a rapidly disseminating viral infection caused by severe acute respiratory syndrome coronavirus 2 (SARS-CoV-2). It has been identified as a member of a large family of enveloped ribonucleic acid (RNA) viruses (Coronaviridae) responsible for causing various respiratory tract infections among humans [1]. It could be a mild upper respiratory tract infection; moderate or severe/critical with clinical signs of pneumonia as that in acute lung injury, ARDS, sepsis, septic shock, and multiple organ dysfunction syndromes (MODS) [2]. The genomic characteristics and phylogenetic network suggest that SARS-CoV-2 is closely related to severe acute respiratory syndrome bat virus (SARS-bat virus). The World Health Organization (WHO) surveillance data of March 18, 2021, reported 140,322,903 laboratory-confirmed cases, with 3,003,794 confirmed deaths globally, and in Pakistan, 16,094 deaths have been reported among 750,158 confirmed cases [3].

This pandemic is impairing both physical and psychological health and wellbeing [4]. The current clinical management and treatment guidelines for COVID-19 focus on prevention, control, and supportive care [5]. The treatment protocol involves oxygen supplementation and mechanical ventilation upon requirement. Only remdesivir has been approved by the Food and Drug Administration (FDA) for the treatment of COVID-19 [6-7]. Furthermore, as per the National Institute of Health (NIH) COVID-19 treatment guidelines, the use of dexamethasone has greatly improved the survival rate among the hospitalized patients requiring oxygen supplementation and/or mechanical ventilation [8-9]. The use of convalescent plasma for hospitalized patients with COVID-19 remains controversial. Emergency use authorization (EUA) was issued on November 30, 2020, by the FDA concerning convalescent plasma administration for treatment without convincing evidence of benefit [10]. Moreover, subsequent publications investigating the role of convalescent plasma have also been inconclusive [11], and no significant survival benefit has been reported so far [12]. A similar conclusion was drawn from the use of convalescent plasma in Pakistan; the Ministry of National Health Services (NHS) declared that plasma therapy should no longer be considered as a cure for coronavirus [13].

Immunocompromised individuals are prone to novel coronavirus infection irrespective of age. According to the existing publications, middle-aged and older individuals between 65 and 85 years are at higher risk of getting infected [14]. China has reported 87% of COVID-19 infection among individuals between 10 and 78 years of age, with the most frequent occurrence among those aged 30-79 years (median age) [15]. The United States is amongst the most seriously affected countries, reporting an age range of 19 to ≥ 85 years, with most cases falling between 65 and 84 years (median age). There is a slight gender-based occurrence susceptibility to this infection, with more males affected as compared to females [16]. The most commonly encountered comorbid conditions among the COVID-19 patients include hypertension, diabetes, cardiovascular disorders (CVDs), and cerebrovascular disorders (CVAs) [17].

Several studies have reported alterations in various biochemical parameters among COVID-19 sufferers throughout the disease course. Lymphocytopenia, thrombocytopenia, leukopenia, high levels of CRP, alanine transaminase (ALT), aspartate aminotransferase (AST), creatine phosphokinase (CPK), and D-dimer are among the laboratory findings that have been recorded among the majority of the COVID-19 clinical studies [18-21]. These variables have been identified as significant clinical predictors of the severity of SARS-CoV-2 viral infection. Moreover, affected patients' chest imaging reveals a spectrum of radiological abnormalities, including bilateral opacities with ill-defined margins, smooth or irregular interlobular septal thickening, air bronchogram, and crazy paving pattern thickening of neighboring pleura.

This study aimed to investigate the clinical and biochemical characteristics of the COVID-19 patients and identify the biological markers and predictors of mortality among these patients. We have also assessed the therapeutic impact of different interventions in terms of disease severity amelioration and outcomes.

## Materials and methods

Study design and participants

This is a case series of 165 patients observed from May 1 to August 31, 2020. A total of 179 PCR-confirmed COVID-19 cases were admitted to OMI Institute during this study period; however, 14 pregnant females were excluded from the analysis, as they were admitted for routine delivery and were either asymptomatic or had mild symptoms. No specific COVID-19-related tests were performed, and no therapeutic assistance was provided to them. The enrolled cases were followed until the day of the last recorded event (in-hospital death or discharge). The COVID-19 severity at the time of admission was evaluated based on clinical parameters and the oxygen requirement. Patients having symptoms consistent with COVID-19 but no hemodynamic compromise, need for oxygen, and normal chest X-ray findings were considered mild. Hypoxia (oxygen saturation (SpO2) <94% but >90%) or chest X-ray with infiltrates involving <50% of lung fields without any complications and manifestations related to the severe condition were categorized as moderate. The severe category of patients included those with clinical signs of pneumonia (fever/cough) together with severe respiratory distress (respiratory rate >30), SpO2 ≤90% on room air, and chest X-ray involving >50% of lung fields. Patients progressing toward complications like ARDS, multiorgan failure, and septic shock were considered critical and treated accordingly.

Biochemical assessments****

Laboratory estimations, including complete blood count (CBC), creatinine, electrolytes, total bilirubin, ALT, AST, and CPK, were assessed daily or every two to three days based on the patient’s condition; CRP, D-dimer, ferritin, LDH, procalcitonin (PCT), absolute lymphocytic count (ALC), and neutrophil-to-lymphocyte ratio (NLR) were measured at baseline and at two-day intervals. Pro-B-type natriuretic peptide (Pro-BNP) was only measured among cases with suspected cardiac failure. Chest X-ray and ECG recordings were examined primarily on admission and then repeated as required depending on the abnormalities observed. An HRCT scan was performed in all patients with dyspnea and SpO2 <94% to monitor the radiological abnormalities. The scans were categorized as grade 1 for those with no chest X-ray findings, grade 2 where the chest X-ray had infiltrates involving <50% of lung fields, grade 3 when the chest X-ray involved >50% of lung fields, and grade 4 for those with bilateral opacities, not fully explained by volume overload, lobar or lung collapse, or nodules.

Data collection

Data were collected using the electronic health records, from baseline (time of admission) to death or discharge. The patient's demographic details, baseline comorbidities, radiographic findings, laboratory findings, COVID-19-associated symptoms, inpatient treatment against COVID-19, disease progression, complications, and outcomes were recorded.

Treatment

Patients were provided supportive care with high-flow oxygen therapy via a nasal cannula or face mask, and voluntary ‘awake prone’ positioning was used for as long as the patient could tolerate. Amongst the drugs used were doxycycline (100 mg BD for five days), ivermectin (150-200 ug/kg/day (on two successive days), famotidine (80 mg/day), remdesivir (200 mg IV on Day 1 followed by 100 mg IV daily on Days 2-5), and steroid (methylprednisolone) (0.5 to 1 mg/kg/day) for 10 days or more depending on the patient’s condition and then tapered off with oral prednisolone over the next two weeks, Tocilizumab in two doses (4 to 8 mg/kg repeated in Q12h. once only). Convalescent plasma was administered among patients who were in the moderate category and progressing or in the severe category. Standard deep vein thrombosis (DVT) prophylaxis with enoxaparin 40-60 mg OD for moderate cases while enoxaparin 40-60 mg Q12h. was used for severe cases. The decision about the possible escalation of treatment was based on initial categorization and day-to-day disease progression. Drug dosage was adjusted among subjects with renal impairment. Remdesivir was not prescribed to patients with ALT >5 times the upper limit of normal.

Statistical analysis

All continuous variables were presented as mean ± standard deviation (SD) or median (interquartile range, IQR) while all categorical variables were given as frequency (n) and percentages (%). The differences in the baseline characteristics, clinical outcome, radiologic scores, biochemical values, and treatments among mild, moderate, and severe/critical COVID-19 cases were assessed through the chi-square test and the student's t-test. A multivariable logistic regression model was used to estimate the predictors. All statistical analyses were conducted using the Statistical Package for the Social Sciences (SPSS) version 22.0 (IBM Corp, Armonk, NY), considering p-value <0.05 significant.

Ethical consideration

The independent ethical review committee of Medicell Institute of Diabetes, Endocrinology & Metabolism (MIDEM) approved the study protocol (reference no. IRB-005/MHS/20; dated: April 27, 2020). Written informed consent was obtained from each patient or the next of kin before inclusion in the study.

## Results

**Figure 1:**
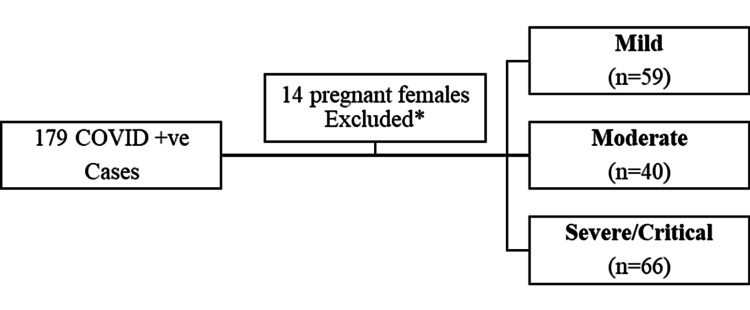
Flow diagram illustrating the inclusion and exclusion of subjects *Due to lack of medical history and records

We collected the inpatient data of 165 COVID +ve cases (Figure 1). The mean age of the cohort was 56.03 ± 15.96 years; 55.1% were males. Electrolyte disturbances, alteration in the blood cell count, and liver function tests were observed among COVID-19 cases in relation to disease severity. The most common comorbid conditions were hypertension and diabetes mellitus, as shown in Table 1.

**Table 1:** Baseline characteristics of the study population *Body Mass Index (BMI); Random Blood Sugar (RBS); Glasgow Coma Scale (GCS); Hemoglobin A1c (HbA1c); Procalcitonin (PCT); Complete Blood Count (CBC); hemoglobin (Hb); White Blood Cell (WBC); Total Bilirubin (T. Bilirubin); Alanine transaminase (ALT); Aspartate transaminase (AST); Creatine Phosphokinase (CPK); Systolic Blood Pressure (SBP); Diastolic Blood Pressure (DBP). **World Health Organization (WHO) Asian BMI Classification (Underweight: <18.5 kg/m^2^, Normal: 18.5-22.9 kg/m^2^, Overweight: 23.0-27.5 kg/m^2^, Obese >27.5 kg/m^2^), $Data representing Inpatient care

Variables		COVID-19 Severity n(%)			Total (n=165)	p-value
		Mild (n=59)	Moderate (n=40)	Severe / Critical (n=66)		
Age; years (mean ± SD)		51.46±19.04	54.40±12.87	61.11±13.16	56.03±15.96	0.002*
Age Groups	< 25 years	6(100)	-	-	6(3.6)	
	25 to 50 years	20(40.81)	17(34.69)	12(24.4)	49(29.7)	
	51 to 75 years	28(29.47)	22(23.15)	45(47.36)	95(57.6)	
	> 75 years	5(33.3)	1(6.66)	9(60)	15(9.1)	
Gender	Male	26(28.6)	23(25.3)	42(46.2)	91(55.1)	0.085
	Female	33(44.6)	17(23.0)	24(32.4.3)	74(44.8)	
Marital Status	Single	6(54.5)	3(27.3)	2(18.2)	11(7.1)	0.333
	Married	52(36.4)	34(23.8)	57(39.9)	143(92.9)	
	Not reported	1(9.09)	3(27.2)	7(63.6)	11(6.66)	
BMI; kg/m^2 ^(mean ± SD)		27.20±4.93	26.77±4.28	27.46±5.51	27.17±4.94	0.908
BMI Categories**	Underweight	1(50)	-	1(50)	2(1.5)	
	Normal	9(39.1)	5(21.7)	9(39.1)	23(17.7)	
	Overweight	9(50)	4(22.2)	5(27.8)	18(13.8)	
	Obese	35(40.2)	25(28.7)	27(31.0)	87(66.9)	
Heart Rate; bpm (mean ± SD)		91.25±11.95	94.72±13.22	93.70±16.08	93.07±14.01	0.445
SBP; mmHg (mean ± SD)		131.27±17.61	135.26±19.43	132.35±21.43	132.65±19.59	0.610
DBP; mmHg (mean ± SD)		78.15±8.93	81.67±8.29	76.76±12.73	78.43±10.62	0.070
RBS; mg/dl (mean ± SD) (N=99)		121.15±38.16	174.85±94.31	164.60±76.79	152.19±73.55	0.009*
Glycemic Categories	Normal	26(44.1)	9(15.3)	24(40.7)	59(59.6)	0.040*
	Pre-diabetes	6(26.1)	5(21.7)	12(52.2)	23(23.2)	
	Diabetes	1(5.9)	6(35.3)	10(58.8)	17(17.2)	
HbA1c (%) (mean ± SD)		6.81±1.87	9.55±3.34	7.33±1.80	7.61±2.42	0.003*
PCT; ng/ml (mean ± SD)		0.16±0.43	0.12±0.23	0.67±1.54	0.35±1.02	0.024*
CBC (mean ± SD)	Hb	12.12±1.74	12.38±2.01	13.83±11.74	12.88±7.64	0.434
	WBC	7.34±4.11	7.47±5.22	10.03±6.43	8.47±5.53	0.015*
	Platelet	237.62±90.04	225.40±118.17	230.03±114.9	231.75±106.71	0.858
	Neutrophils	72.30±10.36	76.26±10.40	81.80±9.63	77.26±10.83	<0.001*
	Lymphocytes	21.42±10.17	18.52±10.21	13.0±9.17	17.17±10.40	<0.001*
Renal & Electrolytes Profile (mean ± SD)	Creatinine	1.16±1.38	0.97±0.45	1.60±2.20	1.30±1.65	0.147
	Calcium	10.56±5.78	8.63±0.49	8.41±0.72	9.02±3.02	0.119
	Sodium	141.15±5.74	140.71±6.01	138.77±5.84	140.09±5.91	0.085
	Bicarbonate	18.31±12.33	20.85±9.58	21.98±8.96	21.95±9.24	0.118
	Potassium	4.09±0.54	4.12±0.57	4.37±0.71	4.21±0.63	0.043*
	Chloride	99.39±8.52	101.47±6.39	99.16±5.04	99.79±6.79	0.253
Liver Function Tests (mean ± SD)	Albumin	3.85±0.49	3.47±0.65	2.71±0.46	3.09±0.67	0.026*
	T. Bilirubin	0.50±0.32	0.52±0.28	0.77±0.94	0.62±0.68	0.086
	ALT	36.75±35.38	42.22±40.12	47.26±53.38	42.64±45.07	0.506
	AST	37.73±32.79	47.96±38.07	50.45±36.58	45.82±35.95	0.201
Health Care Workers^$^	Ward attendant	2(66.7)	1(33.3)	-	3(2.6)	0.332
	Doctor	1(33.3)	1(33.3)	1(33.3)	3(2.6)	
	Staff & Nurses	1(33.3)	1(33.3)	1(33.3)	3(1.81)	
	Ancillary Staff	-	-	1(100)	1(0.60)	
Comorbidities	Yes	33(55.9)	28(71.8)	56(84.8)	117(71.3)	0.002*
	No	26(44.1)	11(28.2)	10(15.2)	47(28.7)	
	Diabetes Mellitus	20(27)	19(25.7)	35(47.3)	74(45.1)	0.088
	Hypertension	22(26.5)	21(25.3)	40(48.2)	83(60.6)	0.300
	Ischemic Heart Disease	8(34.8)	4(17.4)	11(47.8)	23(14.0)	0.650
	Cerebrovascular Disease	1(12.5)	3(37.5)	4(50)	8(4.9)	0.340
	Asthma	2(25)	1(12.5)	5(62.5)	8(4.9)	0.410
	Chronic Kidney Disease	-	-	1(100)	1(0.60)	-
	Other	4(6.7)	5(12.5)	7(10.6)	16(9.6)	0.460

The most prevalent symptoms reported at the time of admission were fever, cough, and breathlessness as shown in Table 2. The majority of the patients in the severe/critical COVID-19 category had established ARDS and sepsis (p<0.05). The outcomes prominently distinguished between mild and severe/critical COVID-19 cases, i.e. 43.5% mild vs. 27.48% severe/critical cases were successfully discharged, whereas 5.8% mild vs. 88.23% severe/critical patients died (p<0.05). An HRCT scan was performed for almost all cases (n=161) except for a few due to temporary mechanical breakdown (Table 2). Out of the 34 patients who died, 15 were on mechanical ventilation, five patients died shortly after arrival, and the remaining had refused invasive ventilatory support.

**Table 2:** Disease characteristics, progression, complications, and outcomes among the studied subjects *Acute Respiratory Distress Syndrome (ARDS), Myocardial Infarction (MI) **Patients reported with mild to moderate Coronavirus Disease (COVID), who later progressed to the severe category or developed serious complications. P-values < 0.05 is statistically significant

Outcome variables		COVID-19 Severity n(%)			Total	p-value
		Mild	Moderate	Severe / Critical		
Symptomatology	Fever	46(24.1)	30(22.2)	59(43.7)	135(75.4)	0.148
	Cough	36(34.3)	22(21.0)	47(44.8)	105(58.7)	0.260
	Sputum	14(63.6)	3(13.6)	5(22.7)	22(12.3)	0.015*
	Runny Nose	3(50)	2(33.3)	1(16.7)	6(3.4)	0.487
	Breathlessness	24(27.3)	15(17.0)	49(55.7)	88(49.2)	<0.001*
	Abdominal Pain	7(41.2)	4(23.5)	6(35.3)	17(9.5)	0.879
	Nausea	9(27.3)	9(27.3)	15(45.5)	33(18.4)	0.507
	Diarrhea	8(38.1)	7(33.3)	6(28.6)	21(11.7)	0.413
	Anosmia	3(42.9)	3(42.9)	1(14.3)	7(3.9)	0.295
	Red Eye	1(50)	-	1(50)	2(1.1)	0.725
	Skin Rash	1(16.7)	1(16.7)	4(66.7)	6(3.4)	0.395
Disease Progression/Complications	ARDS	1(5)**	1(5)**	18(90)	20(12.2)	<0.001*
	Respiratory Failure	1(12.5)**	-	7(87.5)	8(4.9)	0.190
	Sepsis	-	1(7.14)	13(92.8)	14(8.5)	<0.001*
	Coagulopathy	-	1(33.3)	2(66.6)	3(1.8)	0.410
	Heart Failure/MI	-	-	1(100)	1(0.6)	0.470
	Acute Kidney Injury	-	-	2(100)	2(1.2)	0.220
HRCT Scan	Grade 1	56(98.24)	1(1.75)	-	57(34.5)	<0.001*
	Grade 2	-	39(100)	-	39(23.6)	
	Grade 3	-	-	21(100)	21(12.7)	
	Grade 4	-	-	44(100)	44(26.7)	
	Not Done	3(75.5)	-	1(25.0)	4(2.42)	
Outcomes	Discharged	57(43.5)	38(29.0)	36(27.48)	131(79.4)	<0.001*
	Death	2(5.8)	2(5.8)	30(88.23)	34(20.6)	<0.001*

**Figure 2:**
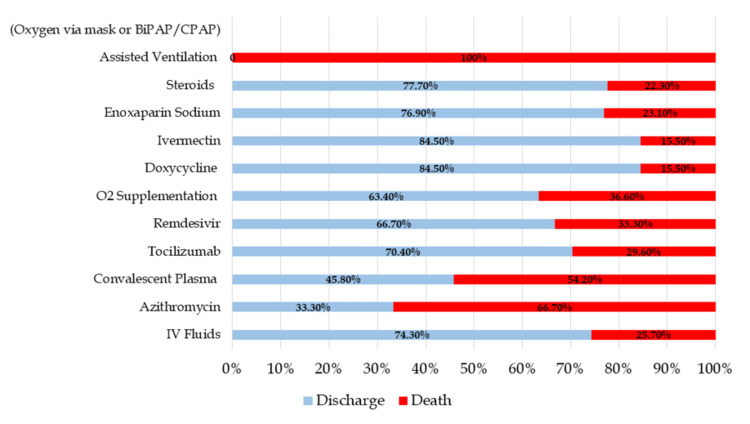
Treatment-based outcomes

These patients were therapeutically managed with one or more of the modalities mentioned above. IV fluids were given to almost all the enrolled patients; 74.3% of these patients were discharged while 25.7% died. The death rate was pronounced among the patients who were already taking azithromycin (66.70%) and who received convalescent plasma (54.20%) while the patients treated with ivermectin, doxycycline, enoxaparin sodium, and steroids had higher discharge rates than death. All patients who received assisted invasive ventilation expired; extracorporeal membrane oxygenation was not available (Figure 2).

The treatment protocol varied in accordance with COVID-19 severity; the requirement of O_2_ supplementation and administration of convalescent plasma was significantly high among the severe/critical COVID-19 cases (p<0.05). Doxycycline and ivermectin were given to 34.5% mild, 29.1% moderate, and 36.4% severe/critical cases while enoxaparin sodium and steroids were given to almost all patients (Table 3).

**Table 3:** Treatment choices based on COVID-19 severity. **Patients reported with mild to moderate COVID, who later progressed to the severe category or developed serious complications. *p-values < 0.05 is statistically significant

Treatment	COVID-19 Severity n(%)			p-value
	Mild (n=59)	Moderate (n=40)	Severe / Critical (n=66)	
IV Fluids	35(32.1)	27(24.8)	47(43.1)	0.365
Azithromycin	-	1(33.3)	2(66.7)	0.419
Convalescent Plasma	4(16.7)	1(4.2)	19(79.2)	<0.001*
Tocilizumab	7(25.9)	2(7.4)	18(66.7)	0.006*
Remdesivir	4(12.1)	12(36.4)	17(51.5)	0.006*
O_2_ Supplementation	20(21.5)	11(11.8)	62(66.7)	<0.001*
Doxycycline	38(34.5)	32(29.1)	40(36.4)	0.109
Ivermectin	38(34.5)	32(29.1)	40(36.4)	0.109
Enoxaparin Sodium	34(29.1)	28(23.9)	55(47.0)	0.007*
Steroids	37(30.6)	29(24.0)	55(45.5)	0.033*
Assisted Ventilation (Oxygen via a mask or BiPAP/CPAP)	1(6.3)**	-	15(93.8)	<0.001*

The SARS-CoV-2 associated markers, including hematological indices, O2 saturation, maximum O2 requirement, inflammatory & biochemical variants, were also studied at baseline, during the hospital stay (midpoint), and at the time of the last recorded event (in-hospital death or discharge) (Table 4). The mean SpO2 at baseline was 91.41 ± 10.9 among discharged patients vs. 80.62 ± 12.7 among deceased patients (p<0.05), which ultimately improved with treatment toward the final outcome 97.06 ± 2.43 amongst those discharged and the deceased patients 88.25 ± 13.22.

**Table 4:** Variation in COVID-19-associated markers w.r.t. disease outcomes (discharged/deceased) *Values are given as median (IQR), n (%), and mean±SD; (-)IQR range cannot be calculated due to data restriction. *p-values < 0.05 is statistically significant. Absolute Lymphocytic Count (ALC); Neutrophil-to-Lymphocyte Ratio (NLR); Lactate Dehydrogenase (LDH); C - Reactive Protein (CRP)

Variables			Outcome		p-value
			Discharged (n=131)	Death (n=34)	
Hematological indices	ALC	Baseline	115400(78875)	97000(70250)	0.063
		Midpoint	117100(114275)	87500(66500)	0.116
		Final outcome	125500(129400)	92800(64200)	0.084
	NLR	Baseline	4.2778(6.45)	10.68(8.19)	<0.001*
		Midpoint	6.3077(5.85)	12.42(10.18)	0.012*
		Final outcome	5.64(6.34)	14.83(13.2)	<0.001*
Inflammatory & biochemical markers	Ferritin	Baseline	383(663.3)	733.35(762.57)	0.057*
		Midpoint	702.5(753.45)	1180(724.45)	0.041*
		Final outcome	619.75(712.25)	1401.15(-)	0.026*
	LDH	Baseline	305(223.5)	568(419)	<0.001*
		Midpoint	343(220.25)	542(350.5)	0.001*
		Final outcome	284(118)	786(819)	<0.001*
	CRP	Baseline	4.5(10)	14.5(17.25)	<0.001*
		Midpoint	1.2(3.6)	9.7(12.07)	<0.001*
		Final outcome	1(2.4)	7.4(21.52)	0.165
	D-Dimer	Baseline	317(530)	526.57(1505.84)	0.158
		Midpoint	507.5(1482)	770(1455)	0.489
		Final outcome	347(488.75)	2260(-)	0.021*
O_2_ saturation		Baseline	91.41±10.9	80.62±12.7	<0.001*
		Midpoint	90.9±6.87	81.22±11.61	<0.001*
		Final outcome	97.06±2.43	88.25±13.22	<0.001*
Maximum O_2_ requirement		1-5 liters	26(44.06)	9(26.4)	0.161
		6-10 liters	17(28.8)	10(29.4)	
		> 10 liters	16(27.11)	15(44.11)	

Significant predictors of death among COVID-19 patients were age (OR 1.05; 95% CI: 1.001-1.109; p=0.047), presence of comorbidity (OR 8.471; 95% CI: 1.941-36.971; p=0.004), raised NLR and LDH in the last recording (OR 1.361; 95% CI: 1.109-1.670; p=0.003 and OR 1.018; 95% CI: 1.001-1.035; p=0.038), and high CRP levels during the hospital stay (OR 1.631; 95% CI: 0.988-2.693; p=0.05) (Table 5).

**Table 5:** Demographic and clinical predictors of outcome (Death) among COVID-19 patients Absolute Lymphocytic Count (ALC); Neutrophil-to-Lymphocyte Ratio (NLR); Lactate Dehydrogenase (LDH); C-Reactive Protein (CRP). *p-values < 0.05 is statistically significant.

Variables		OR	95% CI	p-value
Comorbidities (Present)		8.471	1.941-36.971	0.004*
Age; years (mean ± SD)		1.054	1.001-1.109	0.047*
Male Sex		1.207	0.562-2.593	0.629
BMI; kg/m^2 ^(mean ± SD)		1.09	0.954-1.246	0.204
Health care workers^$^		0.736	0.373-1.452	0.376
ALC	Baseline	1.000	1.000-1.000	0.177
	Midpoint	1.000	1.000-1.000	0.219
	Final outcome	1.000	1.000-1.000	0.114
NLR	Baseline	1.023	0.852-1.228	0.807
	Midpoint	0.901	0.763-1.065	0.221
	Final outcome	1.361	1.109-1.670	0.003*
O_2_ saturation	Baseline	0.982	0.939-1.026	0.419
	Midpoint	0.946	0.895-0.998	0.044*
	Final outcome	0.738	0.628-0.867	<0.001*
CRP	Baseline	0.931	0.83-1.045	0.228
	Midpoint	1.631	0.988-2.693	0.056*
	Final outcome	1.218	0.969-1.532	0.091
Ferritin	Baseline	0.998	0.993-1.004	0.556
	Midpoint	0.998	0.989-1.008	0.702
	Final outcome	1.004	0.995-1.012	0.384
LDH	Baseline	1.005	0.999-1.01	0.088
	Midpoint	0.991	0.977-1.004	0.172
	Final outcome	1.018	1.001-1.035	0.038*
D-Dimer	Baseline	1.009	0.988-1.03	0.413
	Midpoint	0.989	0.962-1.015	0.401
	Final outcome	1.000	0.998-1.002	0.978

COVID-19 tests were repeated for 42 cases, of which 15 were positive; nine of them were later discharged as soon as their condition stabilized while six died during the hospitalization. 

## Discussion

The first wave of COVID-19 cases presented new challenges on a global level for the medical staff and the hospitals, which had never been experienced previously. This new air-borne infectious disease with ease of dissemination and considerable morbidity and mortality, as reported from the original epicenter, Wuhan, required the healthcare systems of all countries to reinvent themselves and prepare for mass-scale hospital admissions. This has been particularly challenging for low-income countries with precarious healthcare systems.

The cases reported in this study are from the first wave of COVID-19 infection when many private hospital facilities were rapidly converted to isolation facilities conduced to treat COVID-19 infections. This case series reaffirms the disease incidence being highest amongst older individuals between 51 and 75 years of age [12,14]. An age-related dysfunction of the immune response presumably increasing the likelihood of infection as well as its severity. Another study from Karachi has also reported the highest SARS-CoV-2 infection rate in a similar age group [22].

The most frequently observed comorbid conditions amongst the studied patients were hypertension and diabetes, consistent with existing literature [15-16]. In keeping with reports from other countries [15-16], we also observed a significant association between the presence of comorbidities and COVID-19 severity (p<0.05). Patients in the severe category were more likely to develop ARDS and sepsis during hospitalization.

COVID-19 patients may present with a spectrum of asymptomatic disease, display mild upper respiratory symptoms with a viral prodrome, or show severe illness characteristics. In the present study, fever, cough, and breathlessness were the most frequently reported symptoms. Cough and fever were also reported as the most common symptoms in COVID-19 patients in China [23]. X-rays were done in all hospitalized patients, showing an almost normal appearance to a range of soft patchy infiltrates, more concentrated at the peripheries and lower zones. However, a CT scan of the chest was much more helpful in categorizing disease severity in our patients (p<0.05). But due to limited diagnostic sensitivity till now, it could only be reserved as a supplementary investigation [18].

Serum calcium was estimated only in patients who complained of muscle cramps and was found to be relatively low amongst patients in the severe category compared to mild or moderate cases, in keeping with another published study [19]. Furthermore, neutrophil, creatinine, ALT, and AST levels were also altered amongst COVID-19 patients in relation to disease severity and outcomes, which has also been reported by other similar studies [19-20]. Additionally, NLR has been recognized as a useful prognostic hematological marker for the early screening of COVID-19 severity [21]. With respect to disease outcome, it was shown that the patients who recovered and were discharged had comparatively normal median NLR as compared to the severe/critical patients who died. The inflammatory markers, including serum ferritin, CRP, LDH, and D-dimer levels, were also elevated amongst the severe category of deceased cases. In parallel to our findings, previous studies have also reported a similar elevation of CRP, D-dimers, LDH, ESR, ferritin, and PCT levels among severe COVID-19 patients admitted to the ICU [20]. Despite prolonged hospital admissions in ICUs and high dependency units, superadded bacterial infections were surprisingly rare, and elevated PCT was seen only in 26.6% of our severe/critical cases while none were observed with a positive pan-culture report. This is contrary to our previous experience of treating non-COVID serious and critical patients in the ICUs, especially those receiving high-dose steroid as well.

The effects of therapeutic interventions were investigated with reference to disease outcomes. Only patients who were already taking azithromycin were allowed to continue on it, and more patients in this group expired, but no inferences can be drawn from this. The use of convalescent plasma was apparently more common in patients who expired. This may simply be related to plasma being administered mostly to those patients who were more seriously ill or deteriorating, rather than a direct causal effect. A Cochrane Systematic Review on convalescent plasma's safety and effectiveness, including 19 studies, showed inconclusive findings [24]. Patients treated with tocilizumab, remdesivir, doxycycline, ivermectin, enoxaparin sodium, and steroid had better outcomes in terms of survival. Tocilizumab and remdesivir appear to be potential therapeutic agents in the moderate/severe category of patients in this study and have also been reported by other groups [6]. Drawing on our previous experience of using steroids in severe, overwhelming sepsis and severe inflammatory diseases, we used steroids in all our moderate category patients who were progressing in severity and those categorized as severe/critical on admission. Preliminary reports from the Chinese experience with COVID-19 helped elucidate an overshoot of the immune response to SARS-CoV-2, by virtue of causing the cytokine release syndrome, which was helpful in determining the use of steroids in these patients. We feel that we may have saved many lives before evidence from a randomized controlled trial (Recovery trial) reported a significant proportional reduction in mortality with dexamethasone treatment among one-third of the ventilated patients as opposed to placebo [8]. Faced with a new infection like SARS-CoV-2, a very important ethical case can be made to use already known drugs that we are familiar with and understand the possible mechanism of benefit. It will always be important to save lives rather than wait for "evidence" before deploying potentially beneficial treatment. This may be one of the factors explaining a reduced fatality rate in Pakistan of 2.19% as compared to some Western countries like the UK (fatality rate 2.90%), where this treatment option was not extensively used [25].

Significant elevation of interleukin 6 (IL-6) in patients with cytokine release syndrome (CRS) was described in early studies reported from China. This formed the basis of using a monoclonal antibody directed against IL-6, tocilizumab, in patients with severe/critical disease. During this study, it was not possible to estimate IL-6 in Karachi. We, therefore, used clinical markers of severity and biochemical markers outlined by NIH, as evidence of CRS to institute tocilizumab in 66.7% of severe category patients who could afford this treatment while 25.9% of mild cases and 7.4% of moderate cases also received this drug because they either progressed to the severe category or demanded this treatment. A recently published study is confirming the use of tocilizumab in the armamentarium of treatment options for SARS-CoV-2 infection to improve survival in terms of discharge and progression to invasive respiratory support in patients with evidence of CRS and severe hypoxia [26].

Early studies from China and, subsequently, a post-mortem report published from Italy confirmed increased coagulability with de-novo thrombosis and embolism as important pathogenetic mechanisms in severe disease [17]. Almost all the patients in this study received anticoagulation therapy. Around 13.9% of the in-hospital COVID-19 patients required ICU care, and the overall mortality rate was 20.6%, while other published studies have reported comparable mortality rates, but the variation in the ICU admission is considerable. Wu et al. reported 21.9% deaths and 26.4% cases requiring ICU care [27]. In contrast, Zhou et al. reported high mortality and ICU admissions (28.3% and 26%, respectively) [28]. Surprisingly, a local study from Lahore reported 8.3% mortality; however, 30.7% of their patients required ICU care [29]. The differences in our study and one from Lahore may be explained by the availability of intensive care beds, as we were working in a relatively small private hospital as opposed to the pooling of data from four large public and private sector hospitals in Lahore.

Among the predictors of mortality, patients with older age and comorbid conditions were 1.054 and 8.471 times more likely to suffer severe COVID-19 outcomes and expire than the younger patients without comorbidities in our study (p<0.05). These findings are in keeping with a systematic review identifying predictors of mortality among COVID-19 patients, showing older age, hypertension, and diabetes mellitus as significant predictors of mortality among COVID-19 patients [30]. SpO2 measurements were consistently low in ICU patients; a significant association was observed between O2 saturation and in-hospital mortality in our cohort (p<0.05), which is also consistent with another published study [30]. Interestingly, despite the extensive lung involvement in SARS-CoV-2 infection, none of the patients were observed with low sodium attributable to Syndrome of Inappropriate Antidiuretic Hormone Secretion (SIADH). Although these patients were clinically stressed, had severe anxiety, and received a high-dose steroid along with anticoagulants, no cases were reported with bleeding from any site.

The limitation of the present study is that it is an observational study with pre-determined interventions in all categories of patients and no comparable control group. Furthermore, a small percentage of continuous data for each laboratory estimate was not available for every patient. Hence, a prospective study, including a large sample of COVID-19 patients, with pre-determined monitoring parameters, laboratory estimations, and treatment options should be designed and conducted locally in Pakistan to provide further insight into the individual patient’s recovery prospects/survival.

## Conclusions

The study identified several alterations in the clinical profile of COVID-19 patients concerning disease severity, affecting prognosis and survival. Variations in the hematological indices, renal function, LFTs, as well as derangements of biomarkers like NLR, CRP, LDH, D-dimer, and ferritin, are potent indicators of COVID-19 severity and mortality. Clinically, the use of tocilizumab, remdesivir, doxycycline, ivermectin, enoxaparin sodium, and steroid were considered potentially useful treatment options in terms of ameliorating COVID-19 severity and promoting recovery.
